# Bioremediation: An Overview on Current Practices, Advances, and New Perspectives in Environmental Pollution Treatment

**DOI:** 10.1155/2017/6327610

**Published:** 2017-11-01

**Authors:** Raluca Maria Hlihor, Maria Gavrilescu, Teresa Tavares, Lidia Favier, Giuseppe Olivieri

**Affiliations:** ^1^Department of Horticultural Technologies, Faculty of Horticulture, “Ion Ionescu de la Brad” University of Agricultural Sciences and Veterinary Medicine, 3 Mihail Sadoveanu Alley, 700490 Iași, Romania; ^2^Department of Environmental Engineering and Management, Faculty of Chemical Engineering and Environmental Protection, “Gheorghe Asachi” Technical University of Iași, 73 Prof. Dr. Docent D. Mangeron Street, 700050 Iași, Romania; ^3^Academy of Romanian Scientists, 54 Splaiul Independentei, 050094 Bucharest, Romania; ^4^Centre of Biological Engineering (CEB), University of Minho, Campus de Gualtar, 4710-057 Braga, Portugal; ^5^Ecole Nationale Supérieure de Chimie de Rennes, CNRS, UMR 6226, 11 Allée de Beaulieu, CS 50837, 35708 Rennes Cedex 7, France; ^6^Department of Chemical, Materials and Industrial Production Engineering, Università degli Studi di Napoli Federico II, Piazzale V. Tecchio 80, 80125 Napoli, Italy; ^7^Bioprocess Engineering Group, Wageningen University and Research, Droevendaalsesteeg 1, 6708 AA Wageningen, Netherlands

Environmental pollution generated the need to search for new environmentally friendly, low-cost, and more efficient environmental clean-up techniques for its removal or reduction. Bioremediation, a branch of environmental biotechnology, is nowadays considered as one of the most promising alternatives. This technology uses the amazing ability of microorganisms or plants to accumulate, detoxify, degrade, or remove environmental contaminants. Bioremediation provides the transformation and/or even removal of organic and inorganic pollutants, even when they are present at low concentration. Continuous efforts are still made to understand the mechanisms by which microorganisms and plants remove or transform environmental pollutants. Thus, the purpose of this special issue was to explore different visions on bioremediation, while addressing recent advances and new ideas in the perspective of efficient process scale-up in view of application at larger scales.

Authors' contributions cover various topics with a range of papers including original research and review articles spanning studies in remediation of different environments which outline new findings in the biotechnology field. This special issue contains five papers including one review article and four original research articles. A brief description of these five manuscripts is detailed below.

During the treatment of wastewater with high ammonium concentrations, as is the effluent originating from anaerobic digestion of pig slurry, the presence of free ammonia (NH_3_ or FA) and/or free nitrous acid (HNO_2_ or FNA) can affect the performance of the partial nitrification process. Thus, in the paper titled “Effect of Free Ammonia, Free Nitrous Acid, and Alkalinity on the Partial Nitrification of Pretreated Pig Slurry, Using an Alternating Oxic/Anoxic SBR” by M. Belmonte et al., the authors applied a strategy allowing the use of organic matter to partially remove nitrite (NO_2_^−^) and nitrate (NO_3_^−^) generated during oxic phases. Stable partial nitrification was achieved during the treatment of the effluent of an anaerobic reactor fed with pig slurry.

In the paper titled “Identification of Multiple Dehalogenase Genes Involved in Tetrachloroethene-to-Ethene Dechlorination in a* Dehalococcoides*-Dominated Enrichment Culture,” M. Ismaeil et al. investigated a* Dehalococcoides*-dominated enrichment culture (designated “YN3”) that dechlorinates tetrachloroethene (PCE) to nontoxic ethene (ETH) with high dechlorination activity. The metagenome of YN3 harbored 18 rdhA genes (designated YN3rdhA1–18) encoding the catalytic subunit of reductive dehalogenase (rdhA), four of which were suggested to be involved in PCE-to-ETH dechlorination based on significant increases in their transcription in response to CE addition. Moreover, metagenome data indicated the presence of three coexisting bacterial species, including novel species of the genus* Bacteroides*, which might promote CE dechlorination by* Dehalococcoides*.

Thirty-one mercury-resistant bacterial strains were isolated from the effluent discharge sites of the SIPCOT industrial area in the paper of K. Saranya et al. titled “Bioremediation of Mercury by* Vibrio fluvialis *Screened from Industrial Effluents.” An interesting outcome of this study was that the strain* V. fluvialis *demonstrated, on one hand, a high bioremediation efficiency in the detoxification of mercury from mobile solutions and, on the other hand, a low resistance against antibiotics. Hence,* V. fluvialis *can be successfully applied as a strain for the ecofriendly removal of mercury.

In the paper titled “Effect of Hydraulic Retention Time on Anaerobic Digestion of Wheat Straw in the Semicontinuous Continuous Stirred-Tank Reactors,” X.-S. Shi et al. selected a range of process parameters such as the biogas production, methane content, pH value, and volatile fatty acids (VFAs) component and demonstrate their influence on Hydraulic Retention Time (HRT) in two operation modes of STR (Stirred-Tank Reactors). In addition, the degradation of cellulose, hemicellulose, and crystalline cellulose in digested wheat straw was also investigated. The obtained results indicated that HRT is an important parameter that affects the performance and stability in the anaerobic digestion of wheat straw.

Recent approaches using low sulfidogenic bioreactors to both remediate and selectively recover metal sulfides from acidic mine drainage are reviewed in the paper of I. Nancucheo et al. The manuscript titled “Recent Developments for Remediating Acidic Mine Waters using Sulfidogenic Bacteria” also highlights the efficiency and drawbacks of these types of treatments for metal recovery and points to future research for enhancing the use of novel acidophilic and acid-tolerant sulfidogenic microorganisms in AMD treatment.

We hope that this collection of papers provides to the readers a valuable scientific source and support addressing current practices, advances, and new perspectives applicable in the treatment of environmental pollution and we hope it can also help specialists in the field of biotechnology towards sustainable scale-up.

